# Evaluation of the Effects of Different Polishing Protocols on the Surface Characterizations of 3D-Printed Acrylic Denture Base Resins: An In Vitro Study

**DOI:** 10.3390/polym15132913

**Published:** 2023-06-30

**Authors:** Yousif A. Al-Dulaijan

**Affiliations:** Department of Substitutive Dental Sciences, College of Dentistry, Imam Abdulrahman Bin Faisal University, P.O. Box 1982, Dammam 31441, Saudi Arabia; yaaldulaijan@iau.edu.sa

**Keywords:** additive manufacturing, chairside kit, SEM, surface roughness, rapid prototype

## Abstract

Chairside polishing kits are an alternative to laboratory polishing techniques. The effects of using a chairside polishing kit on a three-dimensional (3D)-printed acrylic denture base (ADB) have not been reported previously. Thus, this study aimed to evaluate the effects of different chairside polishing techniques on the surface characterizations of ABD, including surface roughness average (Ra), average maximum profile height (Rz), and scanning electron microscopy (SEM) representations. One hundred and twenty disc-shaped specimens were fabricated from one conventional heat-polymerized (HP) ADB resin and two 3D-printed (Asiga (AS) and NextDent (ND)) ADB resins (n = 40 per material). Each group was further divided based on the polishing protocol (n = 10) as follows: conventional polishing protocol (C), microdont chairside polishing kit (M), shofu chairside polishing kit (S), and an unpolished group (U). The Ra and Rz values were measured using an optical profilometer. Two-way ANOVA and post hoc tests were used for data analysis (α = 0.05) at significant levels. In unpolished groups, there was a statistically significant difference between HP-U vs. AS-U and ND-U groups (*p* < 0.0001). For Ra, the lowest values were observed in HP-C, AS-S, and ND-C. While the highest values were shown in all unpolished groups. Within the material, there were statistically significant differences between the three polishing protocols (C, M, and S) vs. unpolished (*p* < 0.0001), while there was no significant between C, M, and S groups (*p* = 0.05). The Rz values had the same pattern as the Ra values. The two chairside polishing kits were comparable to conventional polishing techniques, and they can be recommended for clinical application.

## 1. Introduction

The population of the world is aging rapidly; it will double by 2050 and triple the number of people aged 80 years and older [1]. Despite efforts to prevent edentulism (complete or partial), the demand for restoring missing teeth is growing [2]. The objectives of prosthodontic treatment, whether removable or fixed, are to restore esthetics, masticatory function, speech, and overall quality of life (QoL) [2,3]. For removable prostheses, numerous materials such as wood, ivory, bone, gold, and porcelain have been used to construct denture bases [4]. However, these materials have several disadvantages, including instability and distortion, the difficulty of fabrication, hygienic and contamination concerns, and unpleasant esthetics [4]. As a result, multiple efforts have been directed to establish desirable materials, such as dental acrylic resins.

Poly-methyl methacrylate (PMMA), a type of dental acrylic resin, has been widely applied in prosthodontics and orthodontics fields for the fabrication of various designs of dental prostheses [5,6,7,8]. The use of an PMMA acrylic denture base (ADB) is widespread due to biocompatibility, ease of fabrication, pleasant esthetics, lighter weight, and relatively low cost [4,9,10,11]. Despite these advantages, PMMA has limitations such as porosity, poor surface properties, poor mechanical strength, discoloration over time, and possible hypersensitivity reactions [12,13,14]. Some of these deficiencies can be managed via the proper polishing of the ADB; however, if ignored, they can cause serious denture-related conditions, such as denture stomatitis and trauma to the underlying tissues [15,16,17]. Other limitations, such as porosity and allergic reactions, can be overcome via computer-aided designed–computer-aided manufacture (CAD-CAM) technology for denture base fabrication [18,19].

A dental prosthesis can be prepared using CAD-CAM via two methods: subtractive manufacturing, where a milling device cuts PMMA blocks to create the prosthesis, and additive manufacturing, where acrylic resin is 3D-printed to fabricate the prosthesis [20,21]. In the literature, studies have reported the comparable surface roughness values of milled PMMA ADBs to heat-polymerized (HP) ADB [22,23]. However, several studies have shown that 3D-printed ADB displays similar or higher surface roughness values compared to HP ADB [24,25,26,27,28,29]. This can be attributed to the material composition and printing parameters [24].

In daily clinical practice, the ADB of a removable appliance is susceptible to corrective grinding and trimming, which can result in a rougher surface [7,30]. Therefore, it is recommended to polish the denture surface to gradually remove rough layers, resulting in a smoother surface and reduction in the surface free energy. This will lead to improved prosthesis hygiene due to less microbial adherence and colonization [3,31].

Conventionally, the adjusted part of the ADB is polished with pumice slurry followed by the use of aluminum oxide (Al_2_O_3_) polishing paste in a laboratory setting [7,30,32]. Efforts have been made to develop several chairside polishing kits for the immediate use after the clinical adjustments of removable dentures and occlusal, or orthodontic appliances. It has been reported that while laboratory polishing produces the smoothest surface, the clinical application of chairside polishing techniques can be a convenient and useful alternative procedure [33].

The impact of various chairside polishing kits on the surface characterization of heat-processed, auto-polymerized, injection-molded, microwave-cured, and CAD-CAM-milled ADBs was evaluated previously [3,7,33,34]. However, no studies have investigated the effect of chairside polishing protocols on the surface roughness average (Ra) and average maximum profile height (Rz) of 3D-printed resins. Therefore, the objective of this in vitro study was to analyze the effect of using different chairside polishing protocols on the surface roughness of 3D-printed ABDs. The null hypothesis was that the use of different polishing protocols would produce different surface roughness values.

## 2. Materials and Methods

**Figure 1:**
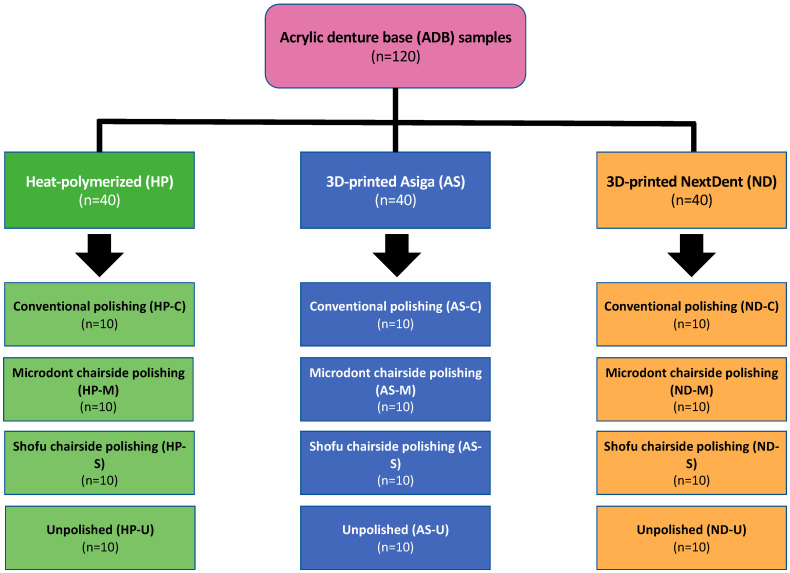
Study flowchart, including specimen distribution over the three different ADB materials and four polishing protocols. n = sample size.

One hundred and twenty disc-shaped specimens (forty samples from each type of ADB material) with dimensions of 10 × 2.5 mm were allocated to twelve subgroups (four subgroups per acrylic resin). The sample size was determined as 10 specimens per group (n = 10) using a power analysis website (www.clincalc.com, accessed on 24 December 2022). The averages and standard deviations (SDs) were acquired from previously published related studies, the power was set as 90% the significant level at 0.05, and the enrollment ration was set at 1 [7,35]. The study flowchart, including groupings and polishing protocols, is presented in Figure 1.

### 2.1. Materials Selection and Specimens Preparation 

Two 3D-printed ADB resins (study groups) and one HP ADB resin (control group) were chosen for this study. For all groups, disc-shaped specimens were planned using CAD software (123D design, Autodesk, version 2.2.14, San Rafael, CA, USA). Afterward, the virtual designs were saved as Standard Tessellation Language (STL) files and transferred into 3D printing systems (ASIGA and NextDent printers). The material composition, printing parameters, and fabrication methods are presented in detail in Table 1.

Before printing, the resin container was homogenized using the LC-3D homogenizer. The exported STL files were transferred to ASIGA MAXTM (ASIGA, Erfurt, Germany) and NextDent 5100 (3D systems, Vertex Dental B.V., Soesterberg, The Netherlands) printers and the specimens were printed according to manufacturer’s recommendations (Table 1) [21,24]. After printing, an isopropyl alcohol (99.9%) was used to clean and remove uncured materials from the printed specimens. The specimens were then post-cued [21,24]. The printing support structures and access materials were removed from the specimens using tungsten carbide burs (AcryPoint No. 0620 bur, Shofu Dental Corp, San Marcos, CA, USA) and low-speed rotary instruments [21,24].

**Figure 2:**
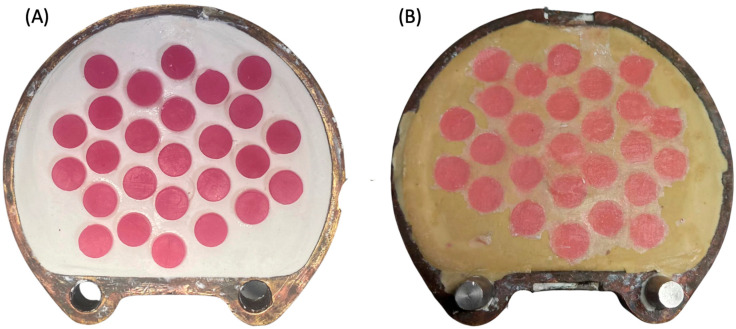
(**A**,**B**) Photo of the heat-polymerization process. (**A**) 3D-printed castable resin placed in the dental plaster during flasking. (**B**) HP specimens after heat processing immediately after deflasking.

Regarding the preparation of HP group specimens, a castable 3D-printed resin (NextDent Cast) was used to replace the wax pattern made from baseplate wax, which is commonly used in heat-cured polymerization to standardize the dimensions of the specimens. Briefly, the same virtual design was transferred to the NextDent 5100 printer to fabricate the specimen patterns using castable resin. The printed disc-shape patterns were invested in dental plaster and dental stone using the conventional flasking procedure (Figure 2A). After 2 h, the printed patterns were removed from the flask and the acrylic resin base material (Major Base) was inserted into the negative molds. The HP group specimens were then processed following the manufacturer’s instructions (Figure 2B).

Prior to the polishing procedures, the specimens underwent finishing using a series of sandpapers (1000 and 2500 grit), followed by the use of tungsten carbide burs on low-speed rotary instruments. Then, all specimens were examined, rinsed, and kept in 37 °C distilled water as described previously [24].

### 2.2. Polishing Protocols 

**Figure 3:**
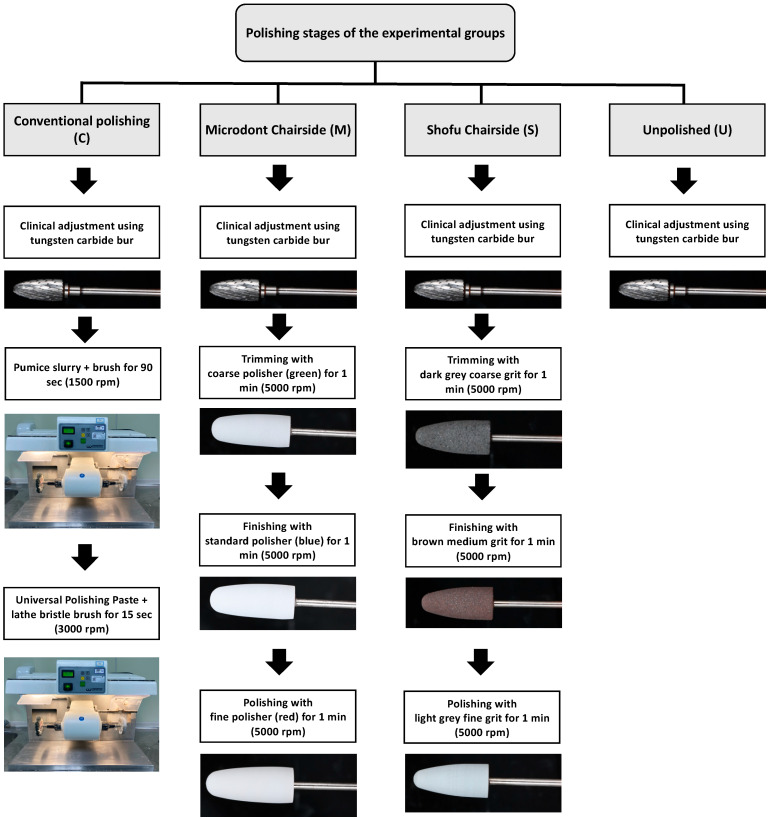
Illustration of the various polishing stages in all groups.

Three polishing groups were tested for each ADB material in this study. One conventional polishing group (control), two groups using chairside polishing techniques (Microdont and Shofu), and the fourth group was left with only finishing (unpolished) to stimulate the clinical adjustment procedure (Figure 1). A summary of the polishing protocols and materials is presented in Table 2, and an illustration of the various polishing stages is presented in Figure 3.

For the conventional polishing groups, the specimens were polished with a pumice slurry (Steribim Super, BEGO GmbH and Co KG; Bremen, Germany) on a brush (Abraso-Soft Acryl, bredent GmbH and Co. KG, Senden, Germany) attached to a grading motors machine (Wassermann WP-Ex 3000, Dental-Maschinen GmbH. Rudorffweg, Hamburg, Germany). This was followed by polishing with a polishing paste (Universal Polishing Paste, Ivoclar Vivadent AG; Schaan, Liechtenstein) using a lathe bristle brush. For the second set of groups, a Microdont chairside polishing kit (Microdont, São Paulo, Brazil) was used. The kit consists of three polishing burs (coarse, standard, and fine polisher). For all third set of groups, a Shofu chairside polishing kit (AcryPoint, Shofu, Dental Corp, Menlo Park, CA, USA) was selected. This kit also uses three different girts (coarse, medium, and fine). The last set of groups were left unpolished to stimulate the clinical adjustment procedure.

**Figure 4:**
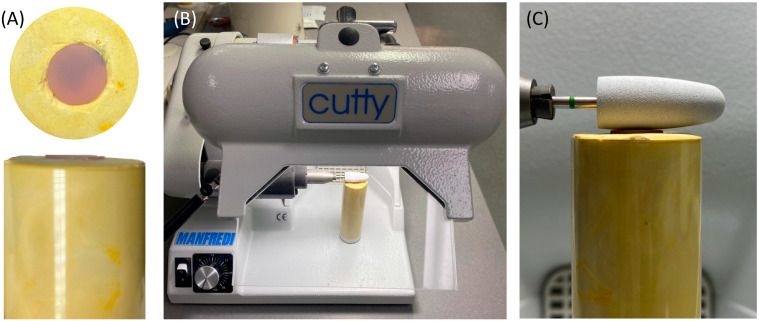
(**A**–**C**) Visualization of the polishing process of the ADB specimens. (**A**) Dental stone mold with secured sample in place. (**B**) Laboratory motor machine. (**C**) Orientation of the polishing bur to the mounted sample.

To standardize the direction and orientation of polishing, the polishing burs were attached to a fixed laboratory motor (Cutty Rapida, Manfredi, Turin, Italy). The ADB specimen was secured in a dental stone mold (Figure 4). In addition, all the polishing procedures were conducted by one operator to control the applied force at a polishing speed of 5000 rpm.

### 2.3. Surface Roughness and Scanning Electron Microscope (SEM) Evaluations

A non-contact optical profilometer (Contour Gt-K1 optical profiler; Bruker Nano, Inc., Tucson, AZ, USA) was utilized to assess the surface roughness values (Ra and Rz) of all specimens. The evaluation involved mounting each specimen on an automated x-y stage and scanning three random scanning points using the previously prescribed method [36]. Afterwards, the capture images underwent analysis to derive the Ra and Rz values of each specimen, and the results were expressed in micrometers (µm) [27].

Prior to SEM evaluation, a single random specimen was selected from each group and was coated with gold using an ion sputter coater (G20, GSEM, Suwon, Republic of Korea). The SEM analysis was conducted using a CUBE-II tabletop machine (EmCrafts Co., Gwangju-si, Republic of Korea) at an accelerating voltage of 10 kV with a secondary electron detector. The micrographs of the HP, AS, and ND groups were captured and analyzed under different magnifications (×100, ×250, ×500, and ×1000) to assess the surface characteristics of the ADB materials. A representative micrograph from all groups was displayed at a magnification of ×1000.

### 2.4. Statistical Analysis 

Statistical analyses were performed. All data are presented as the means ± SD (n = 10). Data normality and distribution were confirmed using the Shapiro-Wilk test. A two-way analysis of variance (ANOVA) and post hoc Tukey tests were used to analyze the data using Sigma Plot 12.0 software (Systat Software Inc., San Jose, CA, USA). Statistical significance was set at a *p*-value of 0.05.

## 3. Results

**Figure 5:**
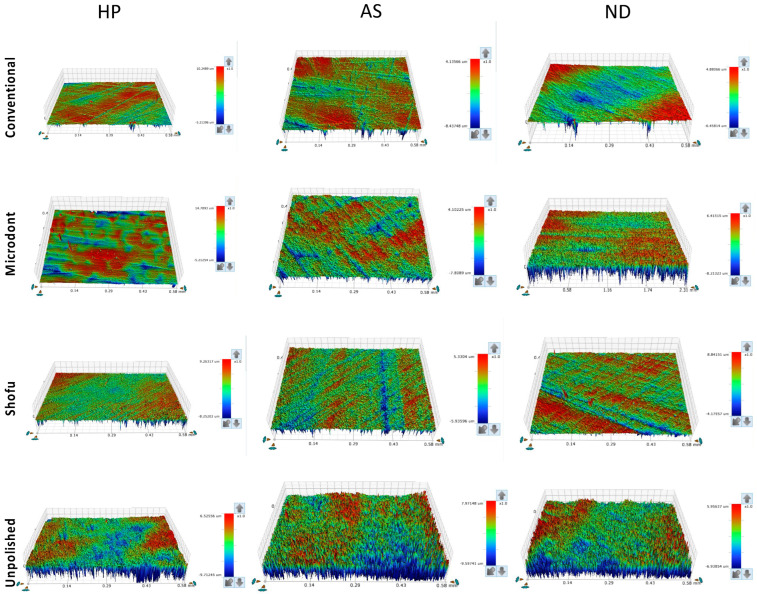
Representative of the scanned specimens for average surface roughness (Ra) of tested groups. HP: heat-polymerized ADBs, AS: Asiga 3D-printed ADBs, and ND: NextDent 3D-printed ADBs.

**Figure 6:**
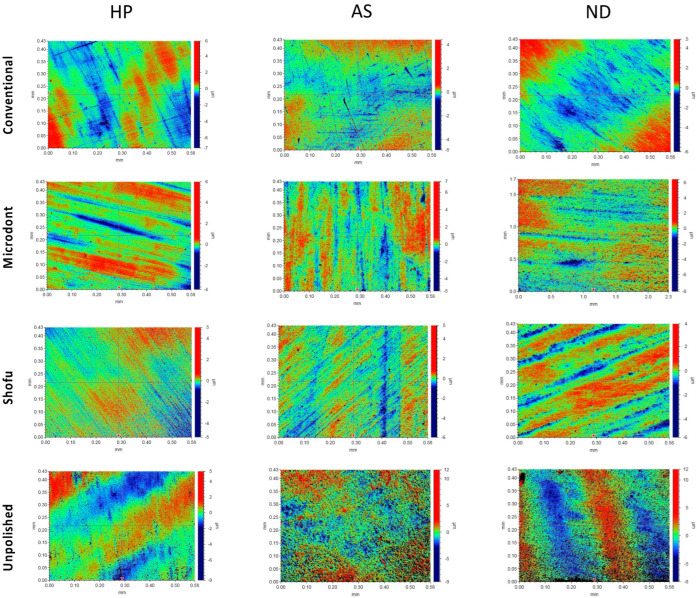
Representative of the scanned samples for average maximum profile height (Rz) of tested groups. HP: heat-polymerized ADBs, AS: Asiga 3D-printed ADBs, and ND: NextDent 3D-printed ADBs.

Figure 5 and Figure 6 show the color parameter representing the average Ra and Rz values, ranging from red (at the top) to blue (at the bottom), with intermediate colors in between. The red areas represent the highest peaks, while the blue areas reveal the deepest valleys. Therefore, the color bar displays the overall smoothness of a surface [37].

**Figure 7:**
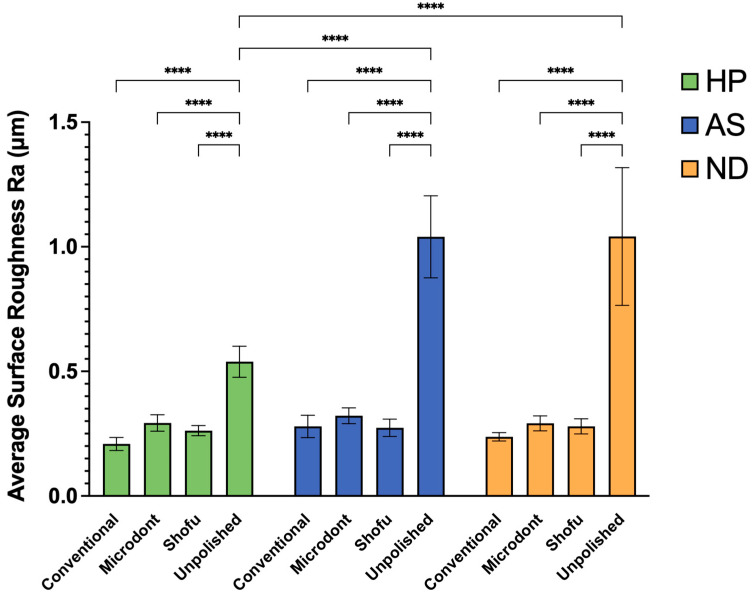
Mean ± SD and the significance of the average surface roughness values (Ra in µm) for the tested groups (level of significance: ****: *p* < 0.0001).

Figure 7 displays the mean and SD of the Ra values for all examined groups. Results from statistical analysis indicate that both the polishing protocol and ADB materials have a significant impact on Ra values (as shown in Table 3). For HP ADB groups, the conventional polishing technique produced the lowest Ra value, followed by the Shofu and Microdont chairside polishing kits. In contrast, the unpolishing group produced the highest Ra value. A two-way ANOVA confirmed that there was a statistically significant difference between the HP-U and three other polishing groups (HP-C, HP-M, and HP-S) (*p* < 0.0001). Similarly, the AS ADB groups showed that the lowest Ra value was achieved using the Shofu chairside polishing kit, followed by the conventional polishing technique and Microdont chairside polishing kit, respectively. Again, like the HP groups, the AS-U group had the highest Ra value. Furthermore, the two-way ANOVA demonstrated a statistically significant difference between the AS-U and three other polishing groups (AS-C, AS-M, and AS-S) (*p* < 0.0001). For ND ABD groups, the Ra values of the three polishing protocols were comparable to the HP groups (ND-C < ND-S < ND-M < ND-U). The two-way ANOVA also showed a statistically significant difference between the ND-U group and all other ND groups (*p* < 0.0001). Finally, comparing the different ADB materials revealed that the HP-U group had a statistically significantly lower Ra value compared to both the AS-U and ND-U groups (*p* < 0.0001).

**Figure 8:**
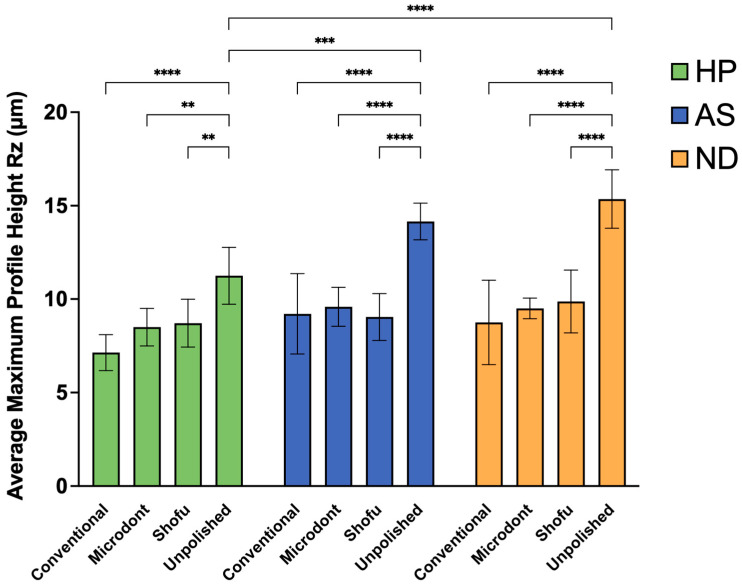
Mean ± SD and the significance of the average maximum profile height values (Rz in µm) for the tested groups (level of significance: **: *p* < 0.01, ***: *p* < 0.001, ****: *p* < 0.0001).

Figure 8 and Table 4 summarize the mean, SD, and the two-way ANOVA statistical analysis of Rz values. Among the HP groups, the lowest Rz value was observed in the HP-C group, followed by HP-M and HP-S group, while HP-U group showed the greatest Rz value. A two-way ANOVA demonstrated statistically significant differences between HP-U and HP-C group (*p* < 0.0001), and slight significant differences between HP-U and HP-M and HP-S groups (*p* < 0.01). Among the AS groups, the lowest Rz values were observed in the AS-S group, followed by AS-C and AS-M groups, while the highest value was observed in the AS-U group. Furthermore, the two-way ANOVA showed statistically significant differences between AS-U and all other AS groups (AS-C, AS-M, and AS-S) (*p* < 0.0001). Regarding the Rz values of the ND groups, the lowest value was observed in the ND-C group, followed by the ND-M and ND-S groups. Similar to HP and AS, the highest value was observed in the ND-U group with a statistically significant difference (*p* < 0.0001). Finally, when comparing the 3D-printed ADB materials (AS and ND groups) to the HP groups, the two-way ANOVA demonstrated significant differences between HP-U and AS-U (*p* < 0.001) and HP-U and ND-U (*p* < 0.0001).

**Figure 9:**
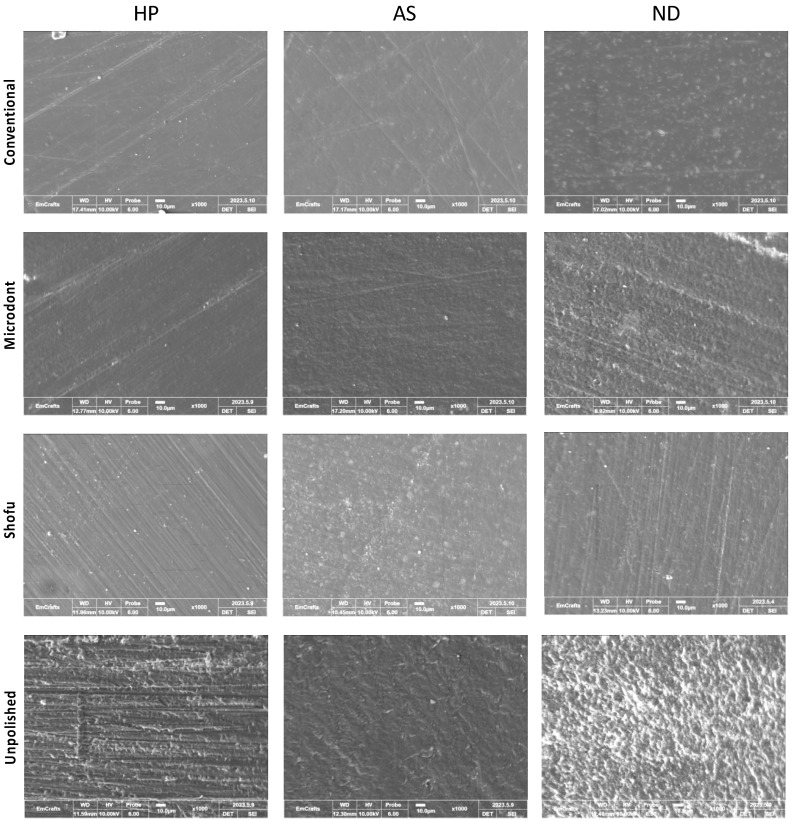
Scanning electron microscope (SEM) images at ×1000 magnification, of the three ADB specimens that were subjected to different polishing protocols.

Figure 9 shows the representative SEM images of one randomly selected specimen of each group at ×1000 magnification. Unpolished specimens showed obvious irregular and rougher surfaces with faint striations in HP, while multiple grooves and depressions were found with ND and AS. The striations in the HP specimens can be attributed to the use of a tungsten carbide bur during the clinical adjustment stimulation. The specimens of 3D-printed resin revealed replicated oblique ridges, which are characteristic of the layer-wise printing method. Furthermore, the surface topography of the polished specimens has been altered, showing different surface features with smooth backgrounds. However, some faint striations with different orientations were observed, which represent the direction of the abrasive particle during polishing protocols.

## 4. Discussion

The longevity of a removable prosthesis depends on the surface roughness of the ADB. Therefore, this research was conducted to investigate the effects of different polishing protocols on the surface characterization of 3D-printed ADBs. Based on the results, the null hypothesis was rejected. The presented results showed that the conventional polishing technique and chairside polishing kits produced comparable surface roughness (Ra and Rz) values regardless of the ADB material. In addition, the unpolished 3D-printed ADB (AS-U and ND-U) showed statistically significant higher Ra and Rz values compared to unpolished heat-polymerized ADB (HP-U).

During prosthodontic procedures, clinical adjustments to the ADB are often required. These adjustments can alter the finishing and polishing of the prosthesis, resulting in a rougher surface [35]. A rougher surface can cause plaque accumulation, discoloration, material degradation, and an increase in patient dissatisfaction [14,27,35,38,39]. Additionally, plaque accumulation has been linked to dental caries and periodontal disease [35]. However, the results of this study show that both conventional laboratory polishing techniques and chairside polishing kits can result in a clinically acceptable smooth surface that can render plaque accumulation and its clinical impacts. Another clinical implication is that chairside polishing kits can be used as a substitute method of choice when laboratory polishing techniques cannot be performed.

Shim et al. compared the surface roughness of unpolished 3D-printed ADB using three different orientations (0°, 45°, and 90°) [38]. The Ra results of this work (AS-U 1.04 ± 0.17 µm and ND-U 1.04 ± 0.28 µm) were comparable to the value of their 45° group (1.09 ± 0.07 µm). Furthermore, all polished groups showed significantly lower values. In another study that evaluated the surface roughness of 3D-printed ADB with the same printing parameters [24], the Ra surface values after polishing with different grits of sandpapers were 0.98 ± 0.13 µm for AS and 0.83 ± 0.13 µm for ND. Comparing their results, the Ra values of all polishing protocols (C, M, and S) in this study were significantly lower.

To the best of auhtor’s knowledge, only one investigation has been published in the literature that evaluated the effects of using JOTA^®^ denture polishing kit (Jota AG, Rüthi, Switzerland) on the surface roughness of 3D-printed ADB [40]. However, only one chairside polishing kit was used in that study [40]. Other studies have been conducted on heat-pressed, auto-polymerized, injection-cured, and CAD-CAM-milled ADBs [3,7,18,33,35]. Their results were in agreement with the present results, showing that the lowest Ra value is associated with conventional laboratory polishing, and that chairside polishing techniques produced comparable results to the conventional procedure.

The surface roughness of heat-cured ADB can be influenced by several factors, including the powder/liquid ratio, proper handling and mixing, and the dental stone texture. In this study, the wax patterns that were commonly used to produce the HP specimens were replaced by 3D-printed castable resin (Figure 2A). While the surface microtexture of printable resin is different than baseplate wax, the results of this study showed that it did not affect the Ra values after polishing. This confirms that the higher roughness values of unpolished 3D-printed ADB (AS-U and ND-U) can be attributed to the nature of the resin and the mechanism of fabrication. Due to the printing nature (layer-by-layer object building), the layering affects the surface properties at the layer interface, forming edge stepwise effects. These edge stepwise effects are considered the main reason for the increase in surface roughness when compared to HP ADB [27].

The results of Ra values of all polished groups demonstrated relatively lower SD. This can be attributed to two main factors: firstm all the polishing procedures were conducted by the same operator, and second, a fixed laboratory motor was utilized during the polishing to control the direction and orientation of polishing burs. However, there was limited control of pressure that was applied to the specimen by the polishing bur. This can be managed by the fabrication of a locked mount that allows the control of the pressure.

Ra and Rz are two commonly utilized parameters for determining the surface roughness of the dental materials [41]. The Ra value represents the average distance and deviation from the mean line between peaks and valleys across the entire surface using an algorithmic measurement within a single sampling length [41,42]. Conversely, Rz is estimated by averaging the vertical space between the highest peak and the lowest valley over five different sampling lengths [41,42]. It is of great important to evaluate and report both values, as there is a strong correlation between surface roughness and microbial adhesion [14,43,44,45,46]. Moreover, rougher surface can be produced from two possible causes: (1) the increase in surface area of the irregularities, which can be evaluated by Ra value, and (2) the depth of these variabilities that can be evaluated by measuring the Rz value.

Only one study has investigated the Rz value of a 3D-printed ADB, in which coating, plashing, and both surface treatments were evaluated [18]. Additionally, limited numbers of studies reported the Rz values of the conventional and CAD-CAM-milled ADB materials [47,48,49]. Sampaio-Fernandes et al. evaluated the Rz values of conventionally polished injectable-cured PMMA and compared them to polypropylene and polyolefin [47]. Their results showed a significantly lower Rz value (0.34 ± 0.049) compared to the HP-C group (7.14 ± 0.96). This was in agreement with another study by Eghtedari et al. [49].

One way to improve the properties of the resin is to cross-link the polymer chains. This prevents the chains from sliding against each other [50]. The HP polymer used in the present study contains ethylene glycol dimethacrylate (EGDMA) as a cross-linking agent, while the UV-light activated 3D-printed resin has substance components, such as bisphenol A-glycidyl dimethacrylate (Bis-GMA) or urethane dimethacrylate (UDMA) [51]. The uncured residual monomer (RM) requires additional post-curing treatment to form the full-structure cross-linking, resulting in the improvement of the properties by achieving ultimate polymerization [52,53,54] Additionally, HP ADB specimens are fabricated using compression molding, which has a high polymerization rate and minimal residual monomer. The present study utilized photo-polymerized resins, which are generally linked with a low degree of conversion and a high level of RM [27,55]. The amount of RM might affect the abrasion resistance of printed specimens, as reported previously [56]. This is due to the inherent material composition and the process of printing layers [56].

Among the factors affecting the surface properties of 3D-printed resin is the printing technology [57]. In the present study, two technologies were implemented: stereo-lithography (SLA) for fabricating the ND specimens and digital light processing (DLP) for fabricating the AS specimens. However, the results of this investigation showed that there was no significant difference in Ra and Rz values between the two 3D-printed materials (AS and ND) after being polished. Therefore, further investigations with different printing parameters and different polishing protocols are required.

The topography of the specimens, as measured by Ra, Rz, and SEM representative images (Figure 5, Figure 6 and Figure 9), confirmed the findings of the unpolished and polished specimens. All unpolished materials had rough surfaces, which became slightly smooth after polishing. Even though there were insignificant changes in the surface features of the polished specimens according to the polishing protocol, the surface roughness was significantly reduced.

One of the possible limitations of this study is the use of a single printing orientation, specifically 45°. Studies have shown that the surface roughness of 3D-printed resin can be affected by different printing orientations [24,38]. Another limitation is the absence of clinical environment stimulations. Dietary solutions, biofilm complexity, and changing the pH and temperature of the oral cavity can alter the surface properties of the 3D-printed resin. Additionally, the disc-shaped specimens used in this study do not resemble the actual form of ADB. This could affect the results of the study, as the surface properties of 3D-printed resin may be different depending on the geometry of the specimen.

Although the in vivo studies [58,59] established a clinically acceptable value for Ra at 0.2 µm, only the conventional polishing technique with pumice of HP ADB specimens produced values that met this standard. Further investigation is required to assess the nature of biofilm accumulation on the 3D-printed ADB and those that were polished with chairside polishing kits. One possible future direction is to evaluate the effect of using these polishing protocols on several different printing parameters, including printing orientation and thickness, post-curing time and conditions, and different printable resins. Another future direction of this study is the evaluation of wettability, contact angle, and water adsorption of 3D-printed ADBs after being polished by a chairside polishing kit. It has been reported that the hydrophobicity of the ADB surface can lead to microbial adhesion [46]. Another future perspective of chairside polishing kits is to evaluate the effects of these polishing protocols used in 3D-printed ADB materials on mechanical properties, such as flexural strength and microhardness as well as optical properties, such as gloss and stainability. Additionally, future studies should consider the biological and mechanical impacts on the longevity and durability of using an ADB with an actual denture geometry in stimulated oral environments.

## 5. Conclusions

It can be concluded that the conventional polishing protocols produced the smoothest surfaces in HP and 3D-printed (AS and ND) ABD materials. Among the ADB materials, there were no significant differences between the chairside polishing kits and the conventional polishing technique. In addition, the unpolished groups of the two 3D-printed ADBs (AS and ND) had higher Ra and Rz values compared to HP ADB. Moreover, the use of chairside polishing kits in the clinical setting can be an efficient and effective technique when access to the dental laboratory is limited.

## Figures and Tables

**Table 1:** Material composition, fabrication methods and printing parameters.

Material	Brand Name	Composition	Fabrication Methods	Printer	Printing Technology and Parameters	Post Printing Procedures
Major (HP)	Major Base.20 (Major Prodotti Dentari Spa, Momcalieri, Italy)	Powder: Methyl methacrylate polymers, Benzoyl peroxide Liquid: MMA, ethylene glycol, dimethacrylate, N,N-dimehyle-p-toluidine, benzophenone-3	Heat polymerization using castable 3D-printed resin	N/A	N/A	N/A
Asiga (AS)	ASIGA DentaBASE (ASIGA, Erfurt, Germany)	7,7,9(or 7,9,9)-trimethyl-4,13-dioxo-3,14-dioxa-5,12-diazahexadecane-1,16-diyl bismethacrylate Tetrahydrofurfuryl methacrylate Diphenyl(2,4,6-trimethylbenzoyl) phosphine oxide	3D-printed using denture base resin	ASIGA MAXTM	LED-based Digital Light Processing (DLP) Layer thickness: 50 μm Printing orientation: 45°	120 min in Asiga Flash (ASIGA, Erfurt, Germany)
NextDent (ND)	NextDent Base; Denture 3D+ (3D systems, Vertex Dental B.V., Soesterberg, The Netherland)	Ethoxylated bisphenol A dimethacrylate, 7,7,9(or 7,9,9)-trimethyl-4,13-dioxo-3,14-dioxa-5,12-diazahexadecane-1,16-diyl bismethacrylate, 2-hydroxyethyl methacrylate, Silicon dioxide, diphenyl(2,4,6-trimethylbenzoyl)phosphine oxide, and titanium oxide	3D-printed using denture base resin	NextDent 5100	Stereolithography (SLA) Layer thickness: 50 μm Printing orientation: 45°	120 min in LC-D Print Box (3D systems, Vertex Dental B.V., Soesterberg, The Netherland)

N/A: Not applicable for heat-polymerized fabrication methods.

**Table 2:** Groupings and polishing protocols used for acrylic denture base materials.

Group Name	Polishing System	Manufacturer	Composition	Protocol	Recommended Speed (rpm)
Conventional (C)	Pumice slurry	Steribim Super, BEGO GmbH and Co KG; Bremen, Germany	Amorphous silica and quartz	I. Polished with the pumice slurry brush attached to a grading motors machine for 90 s II. Polishing with the paste and lathe bristle brush for 15 s	1500
	Universal Polishing Paste	Ivoclar Vivadent AG; Schaan; Liechtenstein	Aluminum oxide (Al_2_O_3_) particles in paste		3000
Microdont (M)	Microdont chairside acrylic polishing kit	Microdont, São Paulo, Brazil	Bonded abrasives in silicone matrix	I. Trimming with coarse polisher (green marked) (No. 10.232.004) for 1 min II. Finishing with standard polisher (blue marked) (No. 10.232.005) for 1 min III. Polishing with fine polisher (red marked) (No. 10.232.006) for 1 min	5000–10,000
Shofu (S)	AcryPoint chairside acrylic polishing kit	Shofu Dental Corp, San Marcos, CA, USA	Bonded abrasives in silicone matrix	I. Trimming with dark grey coarse grit polisher (No. 0426) for 1 min II. Finishing with brown medium grit polisher (No. 0427) for 1 min III. Polishing with light grey fine grit polisher (No. 0428) for 1 min	5000–10,000
Unpolished (U)	Finishing only	Shofu Dental Corp, Menlo Park, CA, USA	N/A	Tungsten carbide cutter (No. 0620) for 15 s to simulate a clinical adjustment	10,000–15,000

N/A: Not applicable.

**Table 3:** Two-way ANOVA for the effect of resin material and polishing protocol on the average surface roughness (Ra) of each ADB.

Source of Variation	Type III Sum of Squares	*df*	Mean Square	*F*-Value	*p*-Value
Resin material	0.565	2	0.283	29.25	<0.001
Polishing protocol	8.194	3	2.731	282.622	<0.001
Resin material * Polishing protocol	1.146	6	0.191	19.762	<0.001
Error	1.044	108	0.00966		
Total	10.949	119	0.092		

* Statistically significant at 0.05 level of significance.

**Table 4:** Two-way ANOVA for the effect of resin material and polishing protocol on the average maximum profile height (Rz) of each ADB.

Source of Variation	Type III Sum of Squares	*df*	Mean Square	*F*-Value	*p*-Value
Resin material	2	87.743	43.872	21.245	<0.001
Polishing protocol	3	503.25	167.75	81.233	<0.001
Resin material * Polishing protocol	6	39.699	6.617	3.204	0.006
Error	108	223.025	2.065		
Total	119	853.717	7.174		

* Statistically significant at 0.05 level of significance.

## Data Availability

Not available.

## Abbreviations

**Abbreviations**	**Description**
3D	Three-dimensional
ANOVA	Analysis of variance
AS	ASIGA 3D-printed resin
Bis-GMA	Bisphenol A-Glycidyl Dimethacrylate
C	Conventional polishing protocol
CAD-CAM	Computer-aided designed and computer-aided manufacturing
DLP	Digital light processing
EGDMA	Ethylene Glycol Dimethacrylate
HP	Heat-polymerized
M	Microdont chairside polishing kit
µm	Micrometers
ND	NextDent 3D-printed resin
PMMA	Poly-methyl Methacrylate
QoL	Quality of life
Ra	Surface roughness average
RM	Residual monomer
Rz	Average maximum profile height
S	Shofu chairside polishing kit
SD	Standard deviations
SEM	Scanning electron microscope
SLA	Stereolithography
STL	Standard Tessellation Language
U	Unpolished
UDMA	Urethane Dimethacrylate
